# First record of *Phyllorhiza* sp. (Cnidaria: Scyphozoa) in a Chinese coastal aquaculture pond

**DOI:** 10.7717/peerj.6191

**Published:** 2019-01-09

**Authors:** Zhijun Dong, André C. Morandini, Agustin Schiariti, Lei Wang, Tingting Sun

**Affiliations:** 1 Muping Coastal Environment Research Station, Yantai Institute of Coastal Zone Research, Chinese Academy of Sciences, Yantai, Shandong, P.R. China; 2 Center for Ocean Mega-Science, Chinese Academy of Sciences, Qingdao, Shandong, P.R. China; 3 Departamento de Zoologia, Instituto de Biociencias, Universidade de São Paulo, São Paulo, Brazil; 4 Centro de Biologia Marinha, Universidade de São Paulo, São Paulo, Brazil; 5 Instituto Nacional de Investigación y Desarrollo Pesquero, Mar del Plata, Buenos Aires, Argentina; 6 Instituto de Investigaciones Marinas y Costeras, Consejo Nacional de Investigaciones Científicas y Técnicas, Mar del Plata, Buenos Aires, Argentina

**Keywords:** Mastigiidae, Coastal ponds, Alien species, Molecular systematics, Jellyfish blooms

## Abstract

**Background:**

It has been suggested that aquaculture ponds on the Chinese coast could act as breeding grounds for scyphozoans. Here, we present the first record of the scyphomedusa *Phyllorhiza* sp. in an aquaculture pond on the coast of the southern Yellow Sea, based on a combination of morphological characteristics and mitochondrial 16S DNA sequence data.

**Methods:**

A field survey was performed on June 29, 2017 in a pond used for culturing the shrimp* Penaeus japonicus*, located in the southern Yellow Sea, China. Jellyfish specimens were collected for morphological and genetic analysis. The morphological characters of the jellyfish specimens were compared to taxonomic literature. Additionally, phylogenetic analysis of the mitochondrial 16S fragments of these specimens were also conducted.

**Results:**

These specimens had the following morphological characters: hemispherical umbrella without scapulets; J-shaped oral arms; a single larger terminal club on each arm; bluish colored with a slightly expanded white tip; and mouthlets present only in the lower half to one-third of each arm. These morphological features of the medusae indicated that the specimens found in the shrimp culture ponds belong to the genus *Phyllorhiza* Agassiz, 1862, but did not match with the description of any of the known species of the genus *Phyllorhiza.* Phylogenetic analyses of the mtDNA 16S regions revealed that these specimens, together with *Phyllorhiza* sp. from Malaysian coastal waters, belong to a sister group of *Phyllorhiza punctata*. Juveniles and ephyrae of *Phyllorhiza* sp. were observed in the aquaculture pond. The mean density of *Phyllorhiza* sp. medusa in the surface water within the pond was estimated to be 0.05 individuals/m^2^.

**Discussion:**

Based on our observations of the gross morphology and molecular data, we state that the specimens collected in the aquaculture pond can be identified as* Phyllorhiza* sp. This is the first record of *Phyllorhiza* sp. in Chinese seas. Large scale dispersal through ballast water or the expansion of jellyfish aquarium exhibitions are possible pathways of invasion, but this needs to be confirmed in further studies.

## Introduction

Blooms of scyphozoan species have been reported in coastal areas worldwide (Purcell, Uye & Lo, 2007; Dong, Liu & Keesing, 2010; Purcell, 2012; Schiariti et al., 2018). These naturally-occurring phenomena have been increasing in frequency and magnitude in some areas, likely in response to various environmental disturbances including biological invasions (Graham & Bayha, 2008; Condon et al., 2012). In some cases, the great numbers reached by these species while blooming cause negative effects on industries such as coastal power plant operations, local fisheries, aquaculture and tourism (Dong, Liu & Keesing, 2010; Purcell, 2012).

Negative interactions of jellyfish species with aquaculture systems appear to be an increasing problem, due to the intensification of these operations in many coastal areas worldwide, including the Yellow Sea of China (Lo et al., 2008; Purcell, Baxter & Fuentes, 2013; Dong et al., 2017). In coastal areas with high anthropogenic pressure, the expansion of artificial construction for aquaculture may provide new large-scale habitats for the settlement and proliferation of scyphozoan polyps, thereby increasing the magnitude of scyphozoan blooms (Lo et al., 2008; Richardson et al., 2009; Dong, Liu & Keesing, 2010; Purcell, 2012; Duarte et al., 2013; Dong et al., 2018). Previous studies have revealed that ephyrae of the moon jellyfish *Aurelia coerulea* form large blooms in the culture ponds of the sea cucumber *Apostichopus japonicus* along the coasts of the Bohai Sea and Yellow Sea, and also that the artificial reefs used in these ponds provide considerable substrates for the settlement and proliferation of *A. coerulea* polyps (Dong et al., 2017; Dong et al., 2018).

Four species of scyphomedusae form frequent and intense blooms in the Bohai Sea and Yellow Sea of China. While blooms of *Rhopilema esculentum* are economically exploited as a food resource (Dong et al., 2009), those formed by *Aurelia coerulea* (formerly mistaken for *A. aurita*), *Cyanea nozakii,* and *Nemopilema nomurai* have proven to be detrimental to fisheries, tourism, and aquaculture (Dong, Liu & Keesing, 2010). The provinces of Liaoning, Hebei Shandong and Jiangsu along the coasts of the Bohai and Yellow Seas are major areas for the aquaculture of the sea cucumber *Apostichopus japonicus*, the crab *Portunus trituberculatus* and the shrimp *Penaeus japonicus*; they comprise approximately 282,583 hectares of coastal area in total (Chinese Bureau of Fisheries, 2016). The expansion of aquaculture ponds in the coastal area of the Bohai and Yellow Seas may act as nursery grounds for the blooming scyphozoans because these coastal ponds, directly influenced by tidal currents, can eventually allow jellyfish planulae that may be present in the seawater to flow into the ponds, and may provide additional suitable substrates for the settlement and proliferation of benthic polyps (Dong et al., 2018).

The genus *Phyllorhiza* was first described by *L. Agassiz (1862)* and belongs to the family Mastigiidae, a family of true jellyfish established by *Stiasny (1920)*. The genus *Phyllorhiza* is comprised of three valid species: *Phyllorhiza punctata* von Lendenfeld, 1884, *Phyllorhiza luzoni* Mayer, 1915, and *Phyllorhiza pacifica* Light, 1921 (Jarms & Morandini, in press). Species of this genus are distributed mostly in Indo-Pacific waters, but none of the valid species have previously been reported in China. As part of a program aiming to investigate scyphomedusae occurrence in aquaculture ponds along the coasts of the Bohai and Yellow Seas, preliminary sampling was conducted at a shrimp culture pond in the southern Yellow Sea, where a mass occurrence of a scyphozoan jellyfish was found by the fisherman. Thus, the goal of the present study is to divulge the data obtained from this survey, reporting the first occurrence of *Phyllorhiza* sp. in Chinese coastal waters. The study is based on a combination of morphological analyses and mtDNA 16S sequence data.

## Material and Methods

**Figure 1:**
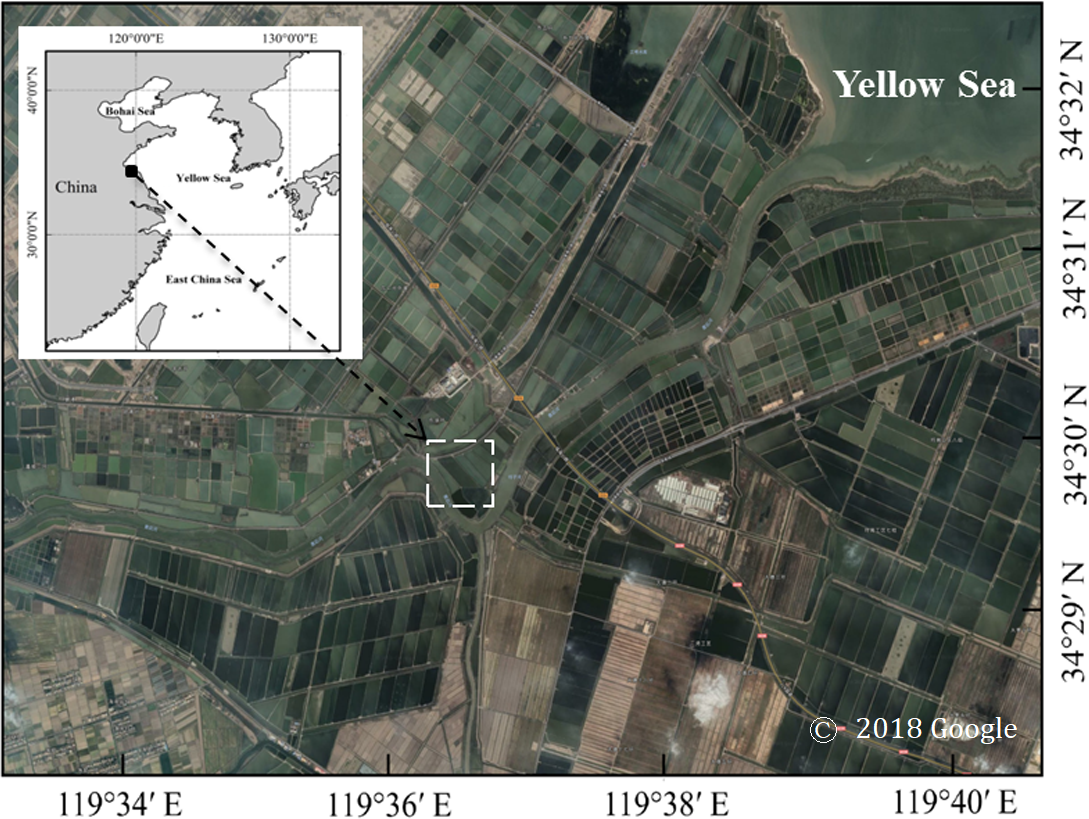
Sampling site for *Phyllorhiza* sp. in a coastal aquaculture pond in the Southern Yellow Sea, China. This figure was generated using Adobe Photoshop CS2 software (URL http://www.adobe.com/), based on data from Google Earth (v.7.1).

**Figure 2:**
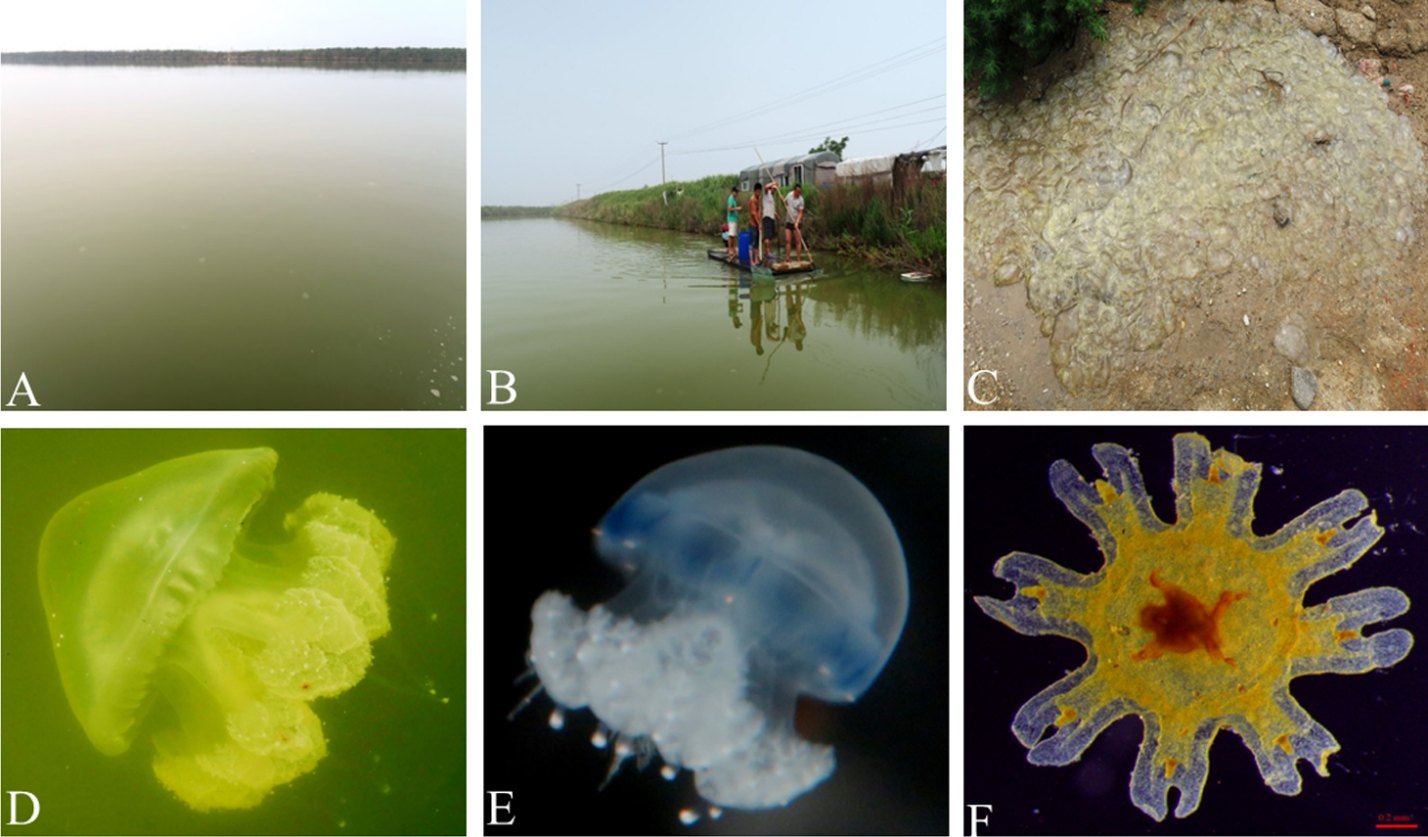
Occurrence of *Phyllorhiza* sp. in a coastal aquaculture pond, Southern Yellow Sea, China. (A) Occurrence of *Phyllorhiza* sp. in the surface water of the coastal aquaculture pond. (B) Fisherman are removing medusae of *Phyllorhiza* sp. using hand nets. (C) Huge number of *Phyllorhiza* sp. medusa were caught. (D) Morphological characters of *Phyllorhiza* sp. Medusa. (E) Morphological characters of *Phyllorhiza* sp. juvenile medusa. (F) Morphological characters of *Phyllorhiza* sp. ephyra.

A field survey was performed between 9:00 to 11:00 AM on June 29 2017 in a *Penaeus japonicus* (Japanese tiger prawn) culture pond. located in the southern Yellow Sea, Jiangsu Province, China (34°29′51″N, 119°36′24″E; Fig. 1). The pond occupies an area of *ca*. 5. 77 ×10^4^ m^2^ and its depth ranges between 1 and 2 m. One intake valve is used to exchange seawater with the adjacent sea twice a month. During our survey the intake valve was closed. Jellyfish were counted visually throughout the entire pond area from a small boat to estimate medusae density (number of observed specimens per m^2^) (Figs. 2A and 2B). The number of medusae in the entire pond area was estimated using a line transect, counting every individual seen 1 m on each side of the line. Visual counts of medusae were carried out during daylight hours, between 9:30 and 10:00 AM. Since turbidity was very high, only the medusae occurring in the upper 30 cm could be observed. A single surface seawater temperature and salinity measurement was taken *in situ* with a YSI-600 multi-parameter water quality sonde (YSI, Yellow Springs, OH).

Fishermen caught the medusae using hand nets to avoid causing damage to the farmed shrimp *P. japonicus* (Figs. 2B and 2C). For morphological measurement, 55 jellyfish specimens were randomly collected using a hand net (mesh size approximately 1 cm) (Figs. 2B and 2C), and measured using a ruler (cm ± 0.1). The morphological identification of the species was based largely on the descriptions provided by Mayer (1910) and Kramp (1961). Because we did not have access to a plankton net, we collected a 20 L surface water sample in a bucket to check for the presence of ephyrae. This sample contained a single ephyra, which was preserved in neutral Lugol’s solution at 2% final concentration. Ephyra measurements were taken in the laboratory following Straehler-pohl, Widmer & Morandini (2011). The central disc diameter (CDD), the lappet stem length (LStL), and the rhopalial lappet length (RLL) of the collected ephyra were measured and photographed using an Olympus SZX10 stereo microscope fitted with an Optec TP510 digital camera.

For molecular analysis, another three adult medusae were preserved in 95% ethanol at −20 °C until DNA extraction. The total genomic DNA was extracted using the TIANamp Marine Animals DNA Kit (TIANGEN). The mitochondrial 16S fragments were amplified using the universal primers 16S-L (GACTGTTTACCAAAAACATA) and 16S-H (CATAATTCAACATCGAGG) following the PCR conditions described in Ender & Schierwater (2003). The PCR products were analyzed using 1.0% agarose gel electrophoresis, stained with Genecolour™ (Biotium, Fremont, CA, USA), and photographed with transmitted illumination. PCR-amplified DNA fragments were purified and sequenced with an ABI 3730 automatic DNA sequencer at Sangon Biotech Co., Ltd (Shanghai, China) using the primers described above. All PCR products were sequenced in both directions to obtain accurate sequences. The DNA sequence fragments were verified, edited and assembled with BioEdit 7.0 (Hall, 2005). The sequences were blasted in NCBI to confirm their identities. Additionally, closely related sequences were obtained from GenBank for phylogenetic analyses. Neighbor joining analysis of 16S data was performed using the K80 model with 1,000 bootstrap replicates. Phylogenetic analyses were conducted with MEGA 5.0 (Ballard & Melvin, 2010).

## Results

**Figure 3:**
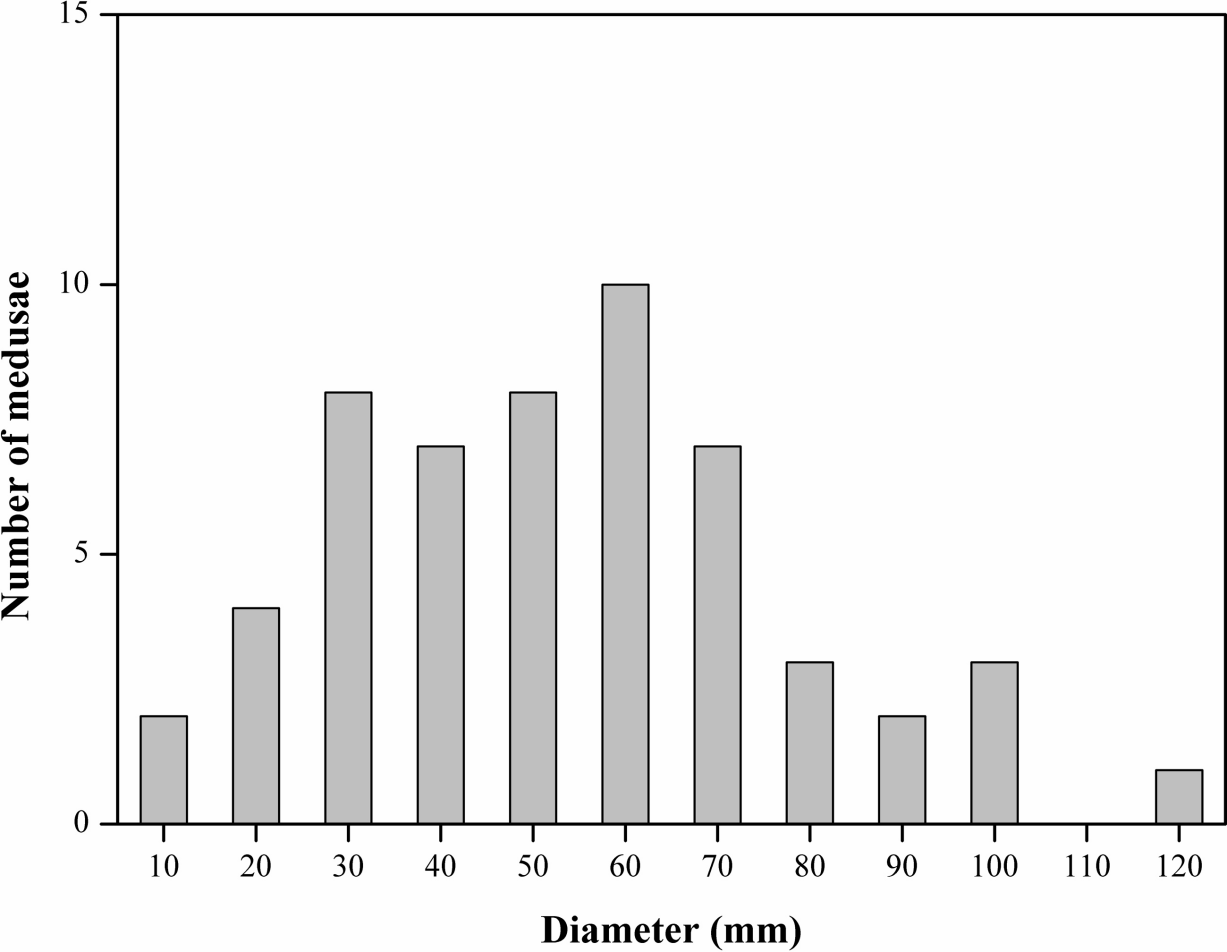
Size distribution (Length, mm) of *Phyllorhiza* sp. of 55 individuals collected in a coastal aquaculture pond, Southern Yellow Sea, China.

Our survey demonstrated the occurrence of the scyphozoan *Phyllorhiza* sp. in an aquaculture pond located on the coast of Jiangsu Province, southern Yellow Sea (Fig. 2A). Jellyfish specimens were clearly Rhizostomeae medusae without scapulets. A total of 2,678 *Phyllorhiza* medusae (including juveniles and adult medusae) were counted in the surface water of the pond, with an estimated density of 0.05 individuals/m^2^. The size-frequency distribution is shown in Fig. 3. On 29 June 2017, the seawater temperature in this aquaculture pond was 28.5 °C and the salinity was 29.5.

The somewhat pointed hemispherical umbrella was between 16 and 120 mm (average: 57 ± 24 mm) in size and was more rounded in smaller specimens. No mature specimens were collected in this study. Larger individuals were translucent (Fig. 2D), while smaller ones had a transparent blue bell margin (Fig. 2E). The oral arms were J-shaped and as long as the bell diameter. They had 3-winged pyramidal mouth arms with naked terminal clubs as appendages and window-like openings at the sides of the mouth arms. There was a characteristic single larger terminal club on each arm, which was bluish colored with a slightly expanded white tip, and mouthlets were present only in the lower half to one-third of each arm. The majority of the medusae were between 30 and 70 mm (average: 57 ± 24 mm).

The collected ephyra had 16 lancet-like rhopalial lappets with an elongated manubrium and was orange brown in colour (Fig. 2F). The CDD, LStL, and RLL of the individual ephyra were 1.23 mm, 0.40 mm, and 0.20 mm, respectively. The features of the medusae and ephyra mentioned above suggest that the specimens found in the shrimp culture ponds might belong to the genus *Phyllorhiza* Agassiz (1862). We have chosen to keep this identification to the generic level, as we cannot clearly identify the species to which the collected animals belong.

**Figure 4:**
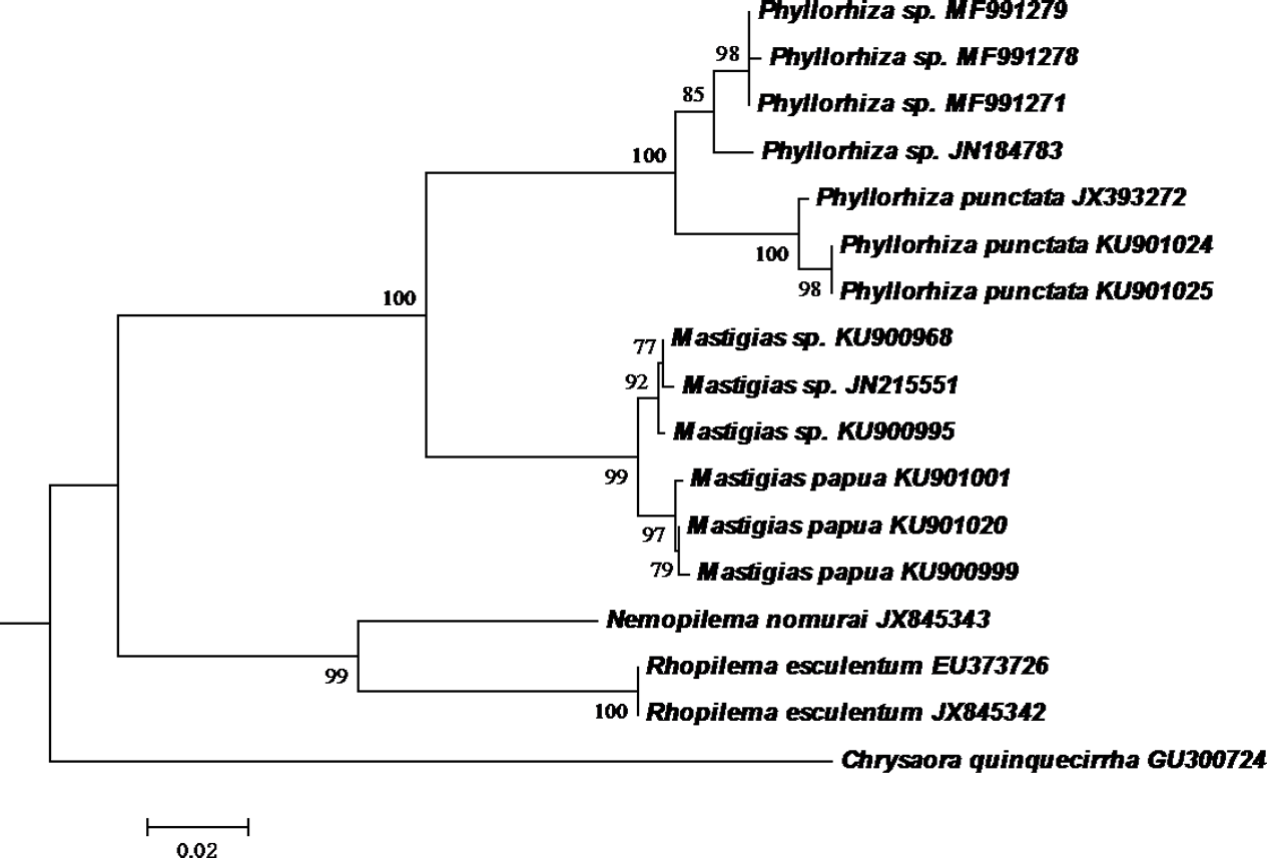
Neighbor-Joining tree for mitochondrial 16S fragments. Bootstrap values higher than 75 are shown above the branches.

The three partial sequences of mitochondrial 16S rDNA genes were 506 bp in length (GenBank accession numbers: MF991271, MF991278, MF991279). A BLAST search of the GenBank database revealed that all of the mtDNA 16S sequences determined in our study nested among the genus *Phyllorhiza*, and were most similar to those of *Phyllorhiza* sp. from the coastal waters of Malaysia (JN184783). The group including our sequences from China and the Malaysian specimen is the sister group of *Phyllorhiza punctata* from Australia and the USA; this grouping is supported by a 100% bootstrap value in neighbour-joining trees (Fig. 4). The genetic distance between *Phyllorhiza* sp. and *P. punctata* calculated using the Tamura-Nei model ranged from 3.8 to 4.8%.

## Discussion

Four species of scyphomedusae frequently occur in the Yellow Sea: *A. coerulea*, *R. esculentum*, *C. nozakii,* and *N. nomurai* (Dong, Liu & Keesing, 2010). However, none of these species were found in this aquaculture pond. Unexpectedly, the scyphomedusae found in this aquaculture pond during this survey appeared to belong to the genus *Phyllorhiza*. This study represents the first record of the scyphozoan *Phyllorhiza* sp. in Chinese seas.

The specimens collected in this study did not match the description of any of the known species of the genus *Phyllorhiza*. The species *P. luzoni* and *P. pacifica* are known to occur in the Philippines, but *P. punctata,* although indigenous to the tropical west Pacific waters of Australia (Graham et al., 2003) is a widespread invasive species occurring in many different places, including southern Brazil, Caribbean Sea, Gulf of Mexico, America, and Mediterranean Sea (Graham et al., 2003; Bolton & Graham, 2004; Haddad & Nogueira Jr, 2006; Deidun et al., 2017). Curiously, a *Phyllorhiza* species has been described for the Chinese coast (South China Sea): *P. chinensis L. Agassiz (1862)*. Mayer (1910) considered it similar to *Cephea cephea*, but other authors (Kramp, 1961) considered it poorly described. Unfortunately, there is no image of *P. chinensis*, and the description is insufficient to determine whether it is a distinct genus or only a different color morph of a known *Phyllorhiza* species. Unfortunately, we did not preserve any specimens for further morphological inspections. However, individuals sampled for molecular analysis clustered in the sister cluster with *Phyllorhiza punctata* sequences available from GenBank, using mitochondrial 16S rDNA data. Based on our observations of the gross morphology and molecular data, we state that the specimens collected in the aquaculture pond can be identified as *Phyllorhiza* sp.

Many scyphozoan species have been confirmed to be invasive species throughout the world (Bayha & Graham, 2014). In general, the dispersal of scyphozoans can occur in two ways: small scale dispersal by physical transport of the pelagic medusa stage and large scale dispersal via transport of polyps and larvae in ships, barges, and offshore drilling platforms (Dawson, Gupta & England, 2005; Bayha & Graham, 2008; Bayha & Graham, 2014). The reasons for the successful invasion events of *Phyllorhiza* sp. are not known. The pond we studied was used for cultivating shrimps with no additional artificial reefs provided, with the exception of the inlets of the pond made of stones. Aquaculture of marine organisms is mostly conducted in coastal areas where water exchange between the coastal ponds and the sea is accomplished by tides or pumps. Generally, farmers perform a water exchange between the aquaculture ponds and the coastal waters twice a month. We infer that the *Phyllorhiza* sp. found in the present study originated from the coastal waters of Southern Yellow Sea.

Ruiz et al. (2000) and Ruesink et al. (2005) indicated that maritime traffic and aquaculture were the main vectors of introduction of alien species in marine environments. Artificial structures in harbors, marinas and aquaculture ponds may provide suitable habitats for alien species and may serve as hot spots for invasions (Ruiz et al., 2000; Ruesink et al., 2005; Forrest, Gardner & Taylor, 2009). In addition, the expansion of jellyfish aquarium exhibitions around the world may enhance the possibility of introductions and invasions, especially because many aquariums are located in coastal waters. *P. punctata* and possible other species in the genus *Phyllorhiza* are cultivated and exhibited in Chinese aquariums. There are two possible invasion pathways of *Phyllorhiza* sp. in the Chinese coastal pond: via maritime traffic or expansion of jellyfish aquariums. Along the coast of Jiangsu Province, there are two huge international ports (Lianyungang Port and Shanghai Port) and one aquarium (Lianyungang Aquarium). The mature medusae may release eggs and larvae, and, if adequate procedures to treat the seawater are not in place, this may allow scyphozoans to colonize coastal waters. In the summer, the Subei coastal currents flow in this region and may potentially transport the planktonic planulae to the pond (Su & Yuan, 2005). Once a scyphozoan population is established (with settled polyps) in a certain coastal pond, the population will expand rapidly under favourable condition. However, further investigations are needed to confirm the occurrence of this species in the study area as a successful invasion.

Introduced jellyfish species are considered an important threat to biodiversity and ecosystem function in marine environments (Bax et al., 2001; Graham et al., 2003). The species *Phyllorhiza punctata* in the Gulf of Mexico is an example of a successful jellyfish invasion. Blooms of this species caused economic losses of up to US $10 million to the shrimp fishery in the Gulf of Mexico in 2000 (Graham et al., 2003). During the survey, fishermen needed to catch the medusae using hand nets to prevent damages to the shrimp *P. japonicus.*

The various sizes of *Phyllorhiza* sp. found in the studied aquaculture pond, and especially the presence of the ephyra, suggest that the species might have established benthic populations in the pond or nearby areas. The accidental finding of a single ephyra suggests that strobilation took place during June. This means that *Phyllorhiza* sp. has found suitable conditions to reproduce, eventually completing its life cycle within the pond or the nearby areas. However, these hypotheses should be tested by inspection for polyps on available substrates, including plastic nets, stones, and woods within the pond or the nearby areas. The presence of only immature medusae, smaller than 120 mm, also suggests that strobilation occurred during spring and summer months, as reported for other Rhizostomeae species (Lucas, Graham & Widmer, 2012). In addition, no mature medusae were found in the pond, as fishermen caught the medusae to avoid damage to the farmed shrimp *P. japonicus*. Continuous monitoring of the occurrence of *Phyllorhiza* sp. population on the studied pond and adjacent areas should be performed to determine whether the species has successfully established a local population and to implement adequate measures to prevent or minimize the eventual problems caused to the shrimp culture in the southern Yellow Sea.

## Conclusions

The scyphozoan jellyfish *Phyllorhiza* sp. has been recorded for the first time in a Chinese shrimp culture pond based on morphological characters and DNA sequence data. The morphological characters of the medusae suggest that the specimens belong to the genus *Phyllorhiza*, but do not match with the description of any of the known species of the genus *Phyllorhiza.* Phylogenetic analyses of the mtDNA 16S regions confirmed that these specimens could be identified as *Phyllorhiza* sp. The invasive vectors of this species in the Chinese coastal pond are not clear. Large scale dispersal through ballast water or the expansion of jellyfish aquarium exhibitions are possible pathways of invasion but this needs to be confirmed in further studies.

##  Supplemental Information

10.7717/peerj.6191/supp-1 Supplemental Information 1 Size distribution (Length, mm) of *Phyllorhiza* sp. of 55 individuals collected in a coastal aquaculture pond, Southern Yellow Sea, China

## Additional Information and Declarations

### Field Study Permissions

The following information was supplied relating to field study approvals (i.e., approving body and any reference numbers): All applicable international, national, and/or institutional guidelines for the care and use of animals were followed.

### DNA Deposition

The following information was supplied regarding the deposition of DNA sequences: The three partial sequences of mitochondrial 16S rDNA genes are accessible via GenBank accession numbers: MF991271, MF991278, MF991279.

### Data Availability

The following information was supplied regarding data availability: The raw data are available in the Supplemental Files.
